# 18F-FDG PET/CT Semiquantitative and Radiomic Features for Assessing Pathologic Axillary Lymph Node Status in Clinical Stage I–III Breast Cancer Patients: A Systematic Review

**DOI:** 10.3390/curroncol32060300

**Published:** 2025-05-23

**Authors:** Anna Hwang, Sana Rashid, Selina Shi, Ciara Blew, Mark Levine, Ashirbani Saha

**Affiliations:** 1 Department of Radiology, McMaster University, 1280 Main Street West, Hamilton, ON L8S 4L8, Canada; hwanga@mcmaster.ca; 2 Department of Radiology, University of Ottawa, 75 Laurier Ave E, Ottawa, ON K1N 6N5, Canada; 3 Department of Radiology, University of Saskatchewan, 105 Administration Pl, Saskatoon, SK S7N 5A2, Canada; 4 University College Dublin School of Medicine, University College Health Sciences Centre, University College Dublin, Belfield, Dublin 4, Ireland; 5 Department of Oncology, McMaster University, 1280 Main Street West, Hamilton, ON L8S 4L8, Canada; 6 Escarpment Cancer Research Institute, McMaster University and Hamilton Health Sciences, 711 Concession Street, Hamilton, ON L8V 1C3, Canada; 7 Centre for Data Science and Digital Health (CREATE), Hamilton Health Sciences, 711 Concession Street, Hamilton, ON L8V 1C3, Canada

**Keywords:** PET/CT, semiquantitative features, radiomics, breast cancer, axillary lymph node metastasis

## Abstract

**Purpose:** To investigate associations between 18F-FDG-PET/CT semiquantitative and radiomic features with pathologic axillary lymph node (ALN) status in stages I–III breast cancer patients. **Methods:** A search was conducted across MEDLINE, EMBASE, and CENTRAL databases. Quality assessment was performed with QUADAS-2 and the radiomics quality score (RQS). Descriptive statistical analysis was performed. **Results:** Most studies were retrospective cohort studies (27/28) and reported only on semiquantitative features (26/28). Most studies were at high risk of bias in patient selection (22/28) and feature extraction (26/28). Semiquantitative features included maximum standardized uptake value (SUVmax), metabolic tumour volume (MTV), and total lesion glycolysis (TLG). Although associations between tumour semiquantitative features and ALN status were reported, the mean/median reported values of tumour SUVmax (3.2–8.6 vs. 2.4–9.4), MTV (2.7–19.2 vs. 1.9–10.5), and TLG (10.6–59.3 vs. 5.6–29.6) in ALN+ vs. ALN− patients were inconsistent between studies. Fourteen studies reported a significantly higher ALN SUVmax in ALN+ patients. Two studies developed models using tumour radiomic features with high accuracy for predicting ALN metastases (81.2% and 80%) but scored low on the RQS. **Conclusions:** Feature-based analysis of PET/CT demonstrates potential for predicting pathologic ALN status in breast cancer patients. However, establishing a clinically meaningful relationship requires higher quality evidence.

## 1. Introduction

In patients with breast cancer, staging of the axilla is important for assessing the extent of locoregional disease and planning treatment. Pathologic staging of the axilla with sentinel lymph node biopsy and/or axillary dissection is often performed, as clinical staging is susceptible to false negatives [1,2,3]. However, surgical exploration of the axilla puts the patient at risk of complications, including lymphedema and pain [4].

Fluorine-18 fluorodeoxyglucose positron emission tomography/computed tomography (18F-FDG PET/CT) is useful in breast cancer staging, particularly in patients with locally advanced disease [5]. Although PET/CT is currently recommended in patients with locally advanced disease for determining axillary nodal burden and occult distant metastases, the recent literature suggests that PET/CT may have a role in earlier stages of disease as well [6]. Although the specificity of PET/CT for axillary nodal detection is high, its sensitivity is only low to moderate, particularly in clinically node negative patients [7,8,9].

Feature-based image analysis has gained traction in the field of medical imaging, with many studies describing the potential of this strategy to improve the diagnosis, prognostication, and management of cancer patients [10,11,12]. Several studies have analyzed PET/CT images using more quantitative methodology, by extracting numerical values or imaging features from a pre-defined region of interest (ROI). These include both semiquantitative features relevant to PET/CT, such as maximum standardized uptake value (SUVmax), metabolic tumour volume (MTV), and total lesion glycolysis (TLG), as well as high throughput (extracted fast and in large numbers) quantitative features, called radiomic features [13,14]. Prior reviews on this subject broadly discuss the potential of PET/CT radiomics in the context of breast cancer for biological characterization, staging, and prognosis [15,16]. However, these studies have not particularly focused on axillary nodal status using histopathology as a reference standard. To address this, we focused on the evidence that PET/CT-derived semiquantitative and radiomic features predicts pathologically confirmed axillary lymph node (ALN) metastases in patients with stage I–III breast cancer in our systematic review.

## 2. Materials and Methods

We adhered to the Preferred Reporting Items for Systematic reviews and Meta-Analysis (PRISMA) guidelines [17]. The study protocol was published on PROSPERO (CRD42024553258). A search strategy was developed in consultation with a medical librarian, using key words such as ‘breast cancer’, ‘axillary’, ‘PET/CT’, ‘radiomics’, ‘semiquantitative’, ‘quantitative’, and ‘texture’. Three databases were searched: MEDLINE (Ovid), EMBASE (Ovid), and CENTRAL (Cochrane). A sample of the literature search strategy can be found in the Supplemental Materials (Supplemental S1). The search included articles, conference papers, and preprints with abstracts written in the English language. No restrictions to the start date were applied, the end date of the search was 30 May 2024, with a goal of completing the systematic review and its reporting within a year, which is the average time reported in the literature [18]. Two reviewers (AH and AS) independently screened the titles and abstracts of all records retrieved from the search, with discrepancies resolved by consensus. Two reviewers (AH and AS) independently reviewed the full text of all records that passed initial screening, with discrepancies resolved by consensus. One reviewer (AH) screened the citations of all included articles and identified two additional articles for full-text review, which was then performed independently by two reviewers (AH and AS).

Our inclusion criteria were as follows: (a) women with newly diagnosed clinical stage I–III breast cancer who underwent standard 18F-FDG-PET/CT for initial staging; (b) sentinel lymph node biopsy (SLNB) and/or axillary lymph node dissection (ALND) were performed without or prior to neoadjuvant therapy; and (c) studies reported on PET/CT semiquantitative or quantitative features in relation to axillary nodal status. Studies were excluded if they included patients who received neoadjuvant therapy prior to axillary surgery, patients who had known distant metastases, patients who did not undergo SLNB/ALND, and patients who did not undergo 18F-FDG-PET/CT for initial staging.

The following data fields were extracted: year, country, inclusion and exclusion criteria, start and end date of data collection, type of study, number of patients, age, type of breast cancer, clinical stage, axillary nodal status, biomarkers, methods of obtaining PET/CT images, ROI, methods of delineating the ROI, types of features extracted, methods of feature extraction, reference standard, mean or median values of features in relation to ALN status, results of receiver-operating characteristics (ROC) analysis, results of univariate or multivariable analysis, and any stated or inferred limitations. Data extraction was performed in Covidence (Veritas Health Innovation, Melbourne, Australia) [19] by two reviewers independently (AH, SR, or SS) with discrepancies resolved by another reviewer (AS). The extracted data were then exported to a Microsoft Excel (version 16.89, Microsoft, Redmond, Washington) spreadsheet.

A tailored QUADAS-2 assessment tool [20] was developed a priori and used to assess the risk of bias and applicability of the included articles, under four domains: patient selection, index test, reference standard, and flow and timing (Supplemental S2). The patient selection domain assessed for inappropriate patient exclusions and whether a random or consecutive group of patients was enrolled. Under this domain, we also examined if the authors clearly specified the clinical stage distribution of the population, which would affect applicability. The index test domain examined if sufficient detail was provided regarding the methodology used for feature extraction, including image acquisition, outlining the region of interest, segmentation, and calculation methods. The reference standard domain assessed if a reliable reference standard was used, namely pathologic confirmation with either SLNB or ALND. The flow and timing domain checked the consistency between patients regarding the time interval between PET/CT imaging and undergoing axillary surgery. Studies were assessed and assigned a high, low, or unclear risk of bias and applicability under the respective domains. Quality assessment was performed by two reviewers independently (AH and CB), with discrepancies resolved by a third reviewer (AS).

The radiomics quality score (RQS) was proposed in 2017 as a standardized method to stratify radiomics studies according to their level of credibility [14]. It assesses 16 methodologic criteria, up to a total of 36 points. The radiomics quality score was applied to the radiomics studies by two authors independently (AH and AS) with discrepancies resolved by consensus.

We performed a descriptive statistical analysis of the data collected. The decision to report descriptively and refrain from meta-analysis was established a priori, as a pilot search demonstrated a lack of standardization between studies regarding image acquisition and methods of feature extraction. Articles were grouped according to the types of features extracted and the ROI. Subgroups of articles at lower risk of bias were identified using the QUADAS-2 tool and analyzed separately. The extracted data were summarized in a tabular format. Where applicable, the median and range of reported values were calculated.

## 3. Results

### 3.1. Search and Eligible Studies

**Figure 1:**
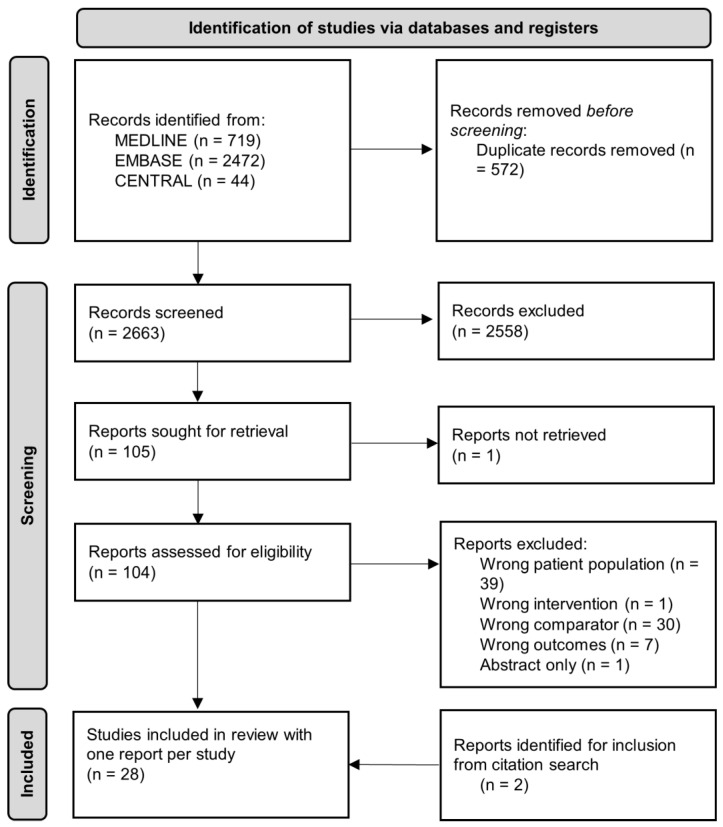
PRISMA flow diagram [17] of all records identified from the literature search.

A total of 2663 records underwent title and abstract screening, after removal of duplicates. Out of these, 105 records were sought for full text review, of which 26 studies met eligibility criteria for inclusion. After a citation search of included articles, an additional two articles were selected for full-text review, both of which met eligibility criteria for inclusion (Figure 1). Studies that investigated dual time point 18F-FDG PET/CT were included, if only feature(s) pertaining to the initial time point, which is equivalent to the standard single-time point PET/CT, were reported. Studies that did not specifically mention SLNB and/or ALND in the text but mentioned that standardized pathologic staging criteria was used were deemed reasonable to be included if the mentioned standardized pathologic staging criteria included SLNB and/or ALND.

A total of 28 studies were included in our systematic review [21,22,23,24,25,26,27,28,29,30,31,32,33,34,35,36,37,38,39,40,41,42,43,44,45,46,47,48]. Several characteristics of included studies are provided in Appendix A (Table A1). Of these, 27/28 were retrospective cohort studies, while one study was prospective [25]. The median population size was 129 patients, ranging from 37 to 671. The most common ROIs explored were the primary breast tumour (22/28) and the axilla or ALNs (16/28). Most included studies (26/28) reported only on semiquantitative features (such as SUV, MTV, or TLG) in relation to axillary nodal status, whereas two studies extracted radiomic features [26,42].

### 3.2. Quality Assessment

**Figure 2:**
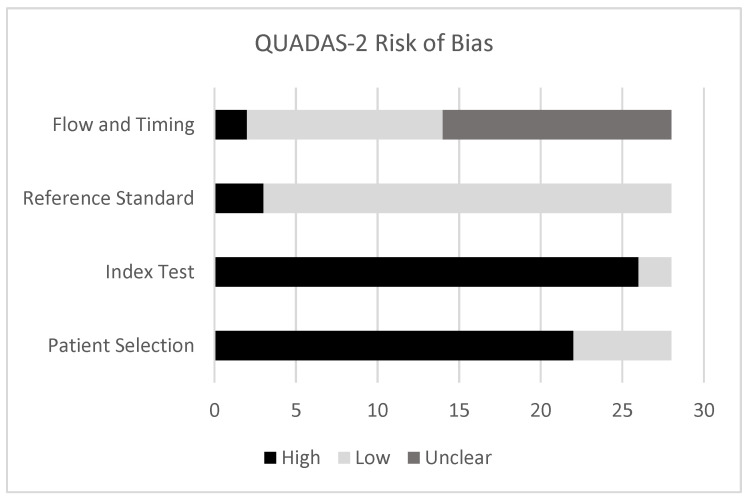
QUADAS-2 risk of bias assessment of included articles [20].

Most of the studies (22/28, 79%) displayed a high risk of bias in the patient selection domain (Figure 2) of the QUADAS-2 [20], most often due to failing to specify whether a consecutive or random sample of patients was enrolled. Other studies excluded participants based on imaging features, for example, low FDG uptake on PET/CT or non-enhancement in magnetic resonance images. Some studies did not specify whether participants received neoadjuvant therapy, which would reduce the credibility of the reference standard [24,25,31,37].

**Figure 3:**
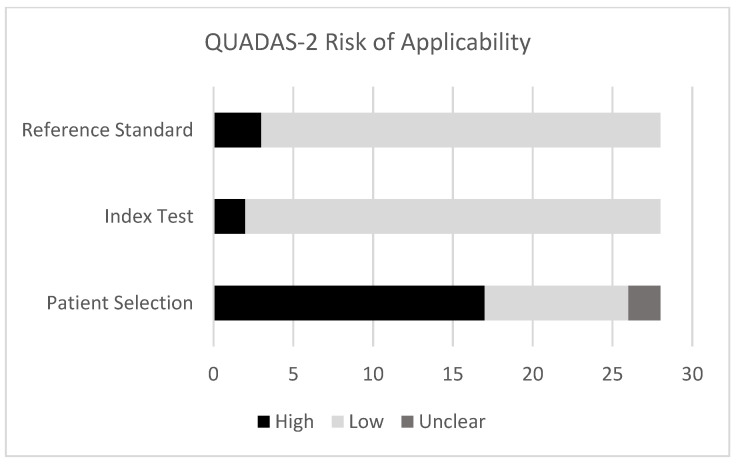
QUADAS-2 risk of applicability assessment of included articles [20].

There was also high concern regarding applicability in the patient selection domain (Figure 3), as many studies did not specify the clinical stage distribution of their patients at all, nor did the authors state that patients with distant metastases were excluded.

Most studies demonstrated a high risk of bias in the extraction of semiquantitative/quantitative features (26/28, 93%). The most common reason for this was that outlining the ROI was often performed by only one observer and intra-rater/inter-rater reliability was not tested, compromising reproducibility. Additionally, several studies did not provide details on how features were calculated. Finally, some studies did not provide any error estimates in their report.

Regarding the reference standard domain, this was strictly adjudicated in the screening process to include only patients who had SLNB and/or ALND; therefore, most studies were at low risk of bias and applicability in this domain. Regarding the flow and timing of the studies, many were at unclear risk of bias as they did not specify the time interval between PET/CT and surgery.

### 3.3. SUVmax of the Primary Tumour

**Figure 4:**
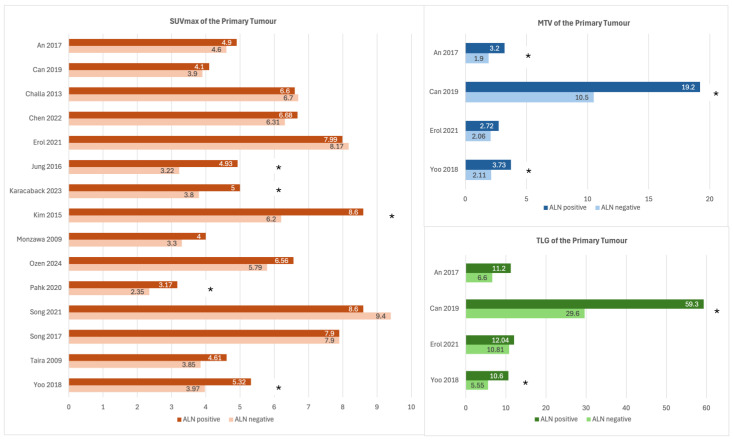
Mean/median values of maximum standardized uptake value (SUVmax), metabolic tumour volume (MTV), and total lesion glycolysis (TLG) of the primary breast tumour in axillary lymph node (ALN)-positive (ALN+ or ALN positive) versus ALN− (ALN negative)patients [21,22,25,26,27,28,29,31,35,36,40,42,43,45,46]. Note that Karan et al. [30] and Kong et al. [32] are not included in the chart of SUVmax, as these studies reported median SUVmax as part of their sub-group analysis for axillary nodal involvement. (*) denotes studies with statistically significant results (*p* < 0.05).

There were 18 studies that investigated tumour SUVmax in relation to ALN status, with varied results (Table 1). Of these, 17 studies reported the mean or median tumour SUVmax in relation to ALN status, while one study reported only the results of ROC analysis. A total of 13 out of the 17 studies (76%) found a higher mean or median SUVmax in ALN+ patients compared to ALN− patients, but only 6 out of 17 (35%) found these results to be statistically significant. There was wide variation in the reported mean or median of tumour SUVmax (Figure 4). The median reported tumour SUVmax of ALN+ cases was 5.3 (range 3.2–8.6), while the median tumour SUVmax of ALN− cases was 4.6 (range 2.4–9.4).

There were five studies that performed multivariable analysis on tumour SUVmax in relation to ALN status [28,31,40,43,46], most (three) of which did not find any statistically significant association with a single cut-off value. However, two studies [28,31] reported a statistically significant odds ratio (odds of the patient being ALN+ versus ALN−) of 3.5 and 4 for SUVmax cutoffs of 4.25 and 2.8, respectively. Although the study by Song et al. [43] did not find a significant general association of tumour SUVmax with axillary nodal status, multiple SUVmax cutoffs determined based on molecular subtypes contributed to the significance of SUVmax with an odds ratio of 4.87.

Of the five [28,29,39,43,46] studies that performed ROC analysis on tumour SUVmax in relation to ALN status, the median optimal cut-off reported for positive nodes was an SUVmax of 3.9 (range 1.8–4.05), producing a median area under the receiver operating characteristics curve (AUC) of 63.6% (range 59.7–84.7%).

None of the studies investigating SUVmax of the primary tumour was at low risk of bias in all QUADAS-2 domains. A total of 5 out of 18 articles were at low risk of bias in the patient selection domain, but not in the index test domain [28,35,39,43,46]. Of these, three out of five reported a significant relationship between SUVmax of the primary tumour and axillary lymph node status. The median value of SUVmax in ALN+ patients was 5.1 (range 4.0–7.9) and in ALN− patients was 3.6 (range 3.2–7.9) Two studies were at low risk of bias in the index test domain, but not in the patient selection domain [26,30]. Of these, one study reported a statistically significant higher SUVmax in ALN+ patients, while the other did not find any significant difference.

### 3.4. MTV and TLG of the Primary Tumour

Four studies investigated MTV and TLG of the primary tumour in relation to ALN status [21,22,27,46]. Three studies reported a statistically significant higher MTV in ALN+ patients, and two studies reported a statistically significant higher TLG in ALN+ patients (Figure 4). The median MTV value reported for ALN+ patients was 3.5 (range 2.7–19.2) and for ALN− patients was 2.1 (range 1.9–10.5). The median TLG value reported for ALN+ patients was 11.6 (range 10.6–59.3) and for ALN− patients was 8.7 (range 5.6–29.6).

There seemed to be high variation in how MTV and TLG were calculated between studies, with low consistency in reported values. For example, the study by Can et al. [22] reported a wide spread of MTV values ranging from 0.6–1435 cm^3^ and TLG (g/mL x cm^3^) values ranging from 2 to 10,737. Additionally, only one study [22] reported units for MTV and TLG.

Of the two studies that performed multivariable analysis, one study performed the analysis on MTV and the other on TLG, as these features were collinear. An et al. [21] found that for a cutoff of 2.38 for MTV, the odds ratio for ALN positivity was 2.696. Yoo et al. [46] reported that for a TLG cutoff of 5.74, the odds ratio was 17.360.

None of the studies investigating MTV and TLG of the primary tumour were deemed at low risk of bias in the QUADAS-2 index test domain. Yoo et al. [46] was the only study that had a low risk of bias in the patient selection domain. This study reported a significantly different median MTV in ALN+ versus ALN− cases (3.73 vs. 2.11) and a significant difference in median TLG in ALN+ versus ALN− cases (10.6 vs. 5.55).

### 3.5. SUVmax of ALNs

All 14 studies reporting on the relationship of SUVmax of ALN to the presence of ALN metastases reported that a higher SUVmax was associated with positive ALNs (Appendix A, Table A2). Studies (four in total) [23,29,37,47] reporting a mean or median value of lymph node SUVmax in relation to ALN status all reported significant differences between node positive and node negative groups. The median value of SUVmax for positive axillary nodal metastases was 5.45 (range 2.2–6.3) in these studies compared to 1.6 (range 1.0–2.79) for negative axillary nodes.

Of the four studies that performed multivariable analysis on SUVmax of lymph nodes in relation to ALN status [28,34,38,44], three found a statistically significant positive odds ratio, the median odds ratio was 14 (range 5.37–15.66) with a median cutoff of 2.5 (0.72–3.2). The other study reported a significant association with ALN metastasis on multivariable analysis, but no further details were specified.

**Figure 5:**
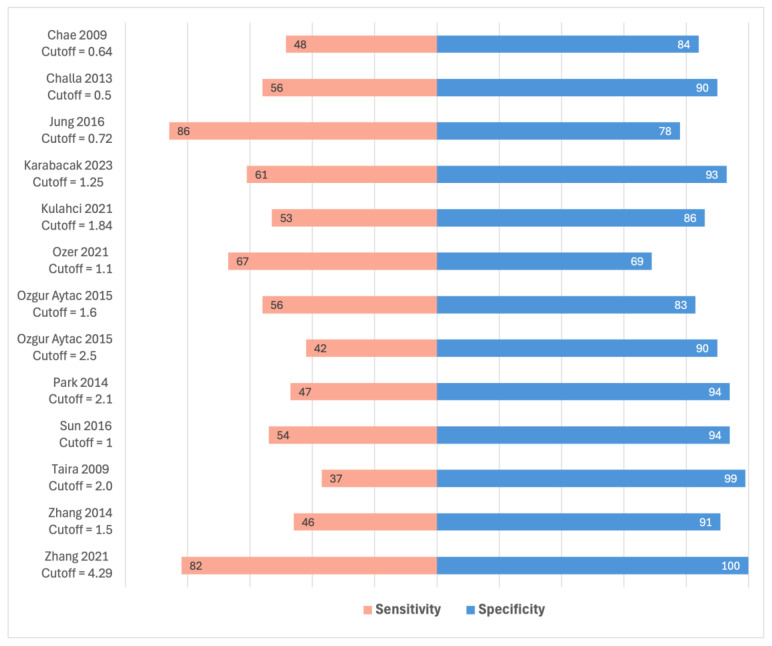
Studies reporting the sensitivity and specificity of axillary lymph node (ALN) maximum standardized uptake value (SUVmax) cut-offs for determining ALN status using receiver operating characteristic analysis [24,25,28,29,33,37,38,41,44,45,47,48].

There were 12 studies that performed ROC analysis, reporting the results of a total of 13 SUVmax cutoff values (Figure 5). The median optimal cut-off for SUVmax of ALN was 1.5 (range 0.5–4.29) with a median sensitivity of 53.8% and a median specificity of 90.5%, suggesting that the SUVmax of ALNs may be a specific but not a sensitive test for ALN status.

One study that differed in design from the others is the study by Zhang et al. [47], in which the authors investigated not individual patients, but individual lymph nodes. Out of 40 patients with stage III N2 disease, they attempted to match the results of 209 surgically resected lymph nodes with the uptake of those individual nodes on PET/CT. The authors reported a high sensitivity of 82.5% and a high specificity of 100% with AUC of 96.1%, with a SUVmax cutoff of 4.29. However, it is not clear how they matched the results of individual dissected lymph nodes with the individual nodal uptake on PET/CT, especially since the study was retrospective in nature.

None of the studies that reported on SUVmax of ALN was deemed at low risk of bias in the QUADAS-2 index test domain. Two studies were at low risk of bias in the patient selection domain [28,44]. Jung et al. [28] reported a sensitivity of 86% and specificity of 78% for detecting ALN metastases using an ALN SUVmax cutoff of 0.72. Sun et al. [44] reported a sensitivity of 54% and a specificity of 94% with an ALN SUVmax cutoff of 1.0.

### 3.6. Semiquantitative Features from Other ROIs

In the study by Pahk et al. [40], the authors investigated SUVmax using visceral and subcutaneous adipose tissue as the ROI. The authors found a significant difference in the SUVmax ratio of visceral to subcutaneous tissue, with a median ratio of 1.65 in ALN+ patients and 1.56 in ALN− patients. On multivariate analysis, using a cutoff ratio of 1.62, the odds ratio was 5.156 (95% CI 2.041–13.028), demonstrating a positive relationship between SUVmax ratio of visceral to subcutaneous fat and the presence of ALN metastases.

### 3.7. Radiomic Features

There were only two studies that investigated higher-order quantitative features [26,42], the ROI was the primary breast tumour in both cases. Chen et al. [26] identified 14 radiomic features (18 PET features and 6 CT features) that showed importance in predicting ALN metastasis status in 180 patients, out of a set of 3124 investigated features. These features were used to train four different models (Table 2). On ROC analysis, the best of these four models had an estimated accuracy of 81.2% and an AUC of 81.7% for predicting ALN metastases, which is higher than almost all reported values for semiquantitative features, except for the studies by Ozkan et al. [39] and Zhang et al. [47].

Song 2021 et al. [42] used radiomic features to create a machine learning model (Table 2) for predicting ALN metastases. The model was developed on a cohort of 75 patients and tested on a separate cohort of 25 patients, producing an estimated sensitivity of 90.9%, specificity of 71.4%, accuracy of 80%, and AUC of 89.0%. However, the testing cohort was small, which limits the generalizability of these results. The study by Chen et al. [26] scored 31% on the RQS [14], while the study by Song et al. [42] scored 25% on the RQS.

## 4. Discussion

When we began our investigation of PET/CT semiquantitative features and radiomics in breast cancer, the existing literature considered breast cancer broadly [15,16]. Based on our PETABC randomized trial of staging in locally advanced breast cancer [5], we realized that utilizing feature-based analysis of PET/CT for determination of distant metastases would offer limited clinical value, as visual analysis of PET/CT is already superior to traditional imaging modalities in this domain. However, PET/CT has limited sensitivity for determining ALN status in breast cancer patients, particularly in earlier stages of disease [7,8,9]. Currently, there are no standardized imaging criteria for classifying ALN status in breast cancer patients, as there are for classifying breast lesions (BI-RADS) or pulmonary nodules (Lung-RADS) [49,50,51]. Since there are no conventional imaging modalities sensitive enough to accurately detect ALN status in breast cancer patients, especially in early stages of disease, many patients proceed to surgical management with SLNB or ALND [1,2,3,9].

Prior studies conducting feature-based or computer-assisted image analyses to characterize various types of cancer have demonstrated the potential of these techniques to improve the diagnosis and management of cancer patients [10,11,52]. We postulated that management of the axilla in Stage I–III breast cancer patients could be improved if the ability of PET/CT to predict ALN status could be augmented by semiquantitative and radiomic analysis. We elected to perform a systematic review, which uses an analytic framework to identify and analyze evidence for a well-defined clinical topic.

In our review, imaging features were extracted predominantly from the primary breast tumour or the ALNs. Although several studies reported associations of SUVmax, MTV, and TLG of the primary tumour with ALN status, there was substantial variation across studies. In comparison to the semiquantitative features derived from the primary tumour, SUVmax extracted from the ALNs showed better consistency in its relationship with ALN status. However, the degree of association for this feature was similar to the sensitivity and specificity based on visual analysis alone, suggesting that there is limited benefit in calculating the SUVmax of lymph nodes [7,8]. Two studies that considered radiomic features from the primary tumour for predicting ALN status reported promising results [26,42], but these are deemed preliminary in nature as the corresponding radiomics quality scores were low (25% and 31%), albeit mildly above the median score (21%) in the literature [53].

There were no studies meeting our inclusion criteria that considered radiomic features extracted from ALNs. Given that semiquantitative features extracted from ALNs demonstrated a more consistent association with ALN status compared to semiquantitative features extracted from the primary tumour, perhaps exploring radiomic features extracted from ALNs could be a high yield focus of future research.

Most studies included in our systematic review were deemed at high risk of bias in both the patient selection (22/28) and index test (26/28) domain of the QUADAS-2 tool. To account for this bias, we investigated subgroups of patients deemed at low risk of bias in either the patient selection domain or the index test domain. The results obtained from these subgroups did not significantly differ when compared to the overall results. However, none of the studies were at low risk of bias in all the QUADAS-2 domains.

Almost all the studies included in our review were retrospective (except for one [25]). Retrospective studies are limited by selection bias, a lack of control over confounding factors, and an inability to firmly establish a causal relationship [54,55]. Applying strict selection criteria can minimize the bias in retrospective studies. However, many of the included studies applied broad selection criteria and did not specify the distribution of important patient characteristics, such as clinical breast cancer stage.

Additionally, we found that reported values of features, especially semiquantitative features, varied greatly between studies. The calculation of these features is dependent on factors such as body weight, blood glucose levels, spatial resolution of the scanner, radioactivity calibration of the scanner, image reconstruction protocols, and filtration parameters [56,57]. However, most studies reported blood glucose levels that varied, and many studies did not specify all these factors when describing their image acquisition technique. There was also variability in how the ROI was outlined. In most studies, this was performed manually by a single observer without testing of intra-rater or inter-rater reliability. Furthermore, many studies did not specify what details (e.g., formula or algorithm) they used to calculate the semiquantitative features. None of the studies referenced a pre-defined guideline or consensus statement specifying which factors to include to ensure standardization or the standardized reporting of feature extraction, such as those published by the Image Biomarker Standardization Initiative [58,59]. A lack of consistent methodology in calculating features was a pitfall in many of our included studies, likely contributing to the high variation in reported values between studies.

A major factor contributing to the low RQSs (25% and 31%) of the two radiomics studies [26,42] was their retrospective design, resulting in both studies having 10 of 36 points (28%) deducted, in part due to a limited ability to control factors related to image acquisition. In addition, the authors did not report calibration statistics, cut-off analysis, or perform validation using external datasets from other institutions, losing 6/36 (17%) points. Neither study considered non-radiomic features in their models or provided an analysis of potential clinical utility or cost-effectiveness, losing 5/36 (14%) points. The code and datasets used were not made publicly available, losing 4/36 (11%). Chen et al. [26] earned more points by applying a resampling method and performing multiple segmentations to reduce segmentation variability.

Future studies investigating semiquantitative and radiomic features should consider a prospective design. With a prospective design, authors can carefully control for factors that can introduce variability, especially in the image-acquisition phase [56,57,60]. Furthermore, in prospective studies, the prognostic ability of PET-CT features associated with ALN metastases are best assessed through long-term follow-up.

To enhance methodological quality, authors of radiomics studies could refer to the items mentioned in the RQS [14] or consult the scoring tool METRICS by the European Society of Medical Imaging Informatics [61]. They should aim to comply with standardized feature extraction techniques, such as those published by the Image Biomarker Standardization Initiative [58,59]. Radiomics studies also need to consider the entire clinical picture, including non-radiomic features in the analysis and providing an assessment of overall clinical utility and cost-effectiveness, if applicable. Finally, collaboration and open sharing of datasets and models among researchers are essential for validation and for ensuring the robustness and reproducibility of radiomic features. Overall, both the quality of science and the quality of reporting in radiomics studies need to improve, which are distinct but important characteristics of a published research study [62,63,64].

As our preliminary analysis of studies on PET/CT derived imaging features showed heterogeneity, we performed a systematic review with descriptive statistical analysis only and did not consider performing meta-analysis. Although we did not conduct a meta-analysis, our study contributes to the evidence base on commonly used modalities in breast cancer that advocates for standardization and reduction in heterogeneity in feature extraction [65,66]. However, with newer modalities such as hyperspectral imaging, heterogeneity among studies has not been noted [67].

Our study is limited by the keywords used in the search process, but a reverse citation search was performed to mitigate the issue. Also, our review is limited to studies published in English. Only 4% of screened titles and abstracts met the criteria for full-text review, suggesting that our search strategy was broad. After full-text review, approximately 1% of articles met criteria for final inclusion. Several articles were excluded due to inappropriate patient inclusions, such as patients who underwent neo-adjuvant therapy or patients with metastatic disease. Many excluded studies did not compare extracted features to a reliable reference standard, or the reference standard could not be deduced from the text. These findings indicate that improvement in study design and reporting are needed in this field. There are several other radiomics studies in the literature that investigate the association of quantitative features of breast tumour in relation to axillary nodal involvement with mixed results [68,69,70,71]; however, these commonly use a reference standard of clinical stage, which itself is not a reliable measure of true nodal involvement. For this reason, these studies were not included in our review.

In summary, although associations were reported between PET/CT-derived imaging features and pathologic ALN status, the evidence is limited by a general high risk of bias and lack of consistency among the studies. This undermines the ability to establish a strong association between the set of semiquantitative and/or radiomic features with ALN status, even if it is present. This also undermines the establishment of no association, even if it is truly not present. Radiomic features, in particular, showed potential to predict ALN status; however, the evidence is deemed preliminary due to a lack of standardized methodology. Therefore, there is an urgent need from a technology perspective to adhere to reliable and consistent methods in imaging feature extraction for the conduct and reporting of studies. An equally important aspect is related to the design of clinical studies, which requires applying appropriate health research methodologies (e.g., prospective well-controlled studies of sufficient sample size) to improve the quality of the evidence.

## 5. Conclusions

Imaging feature-based analysis of PET/CT in breast cancer patients, particularly radiomic analysis, demonstrates potential for predicting pathologically confirmed axillary lymph node status, however the evidence is still in its preliminary stages. The existing literature is limited by a lack of standardization regarding the technical aspects of feature extraction and suboptimal methodological design. Future studies should focus on developing and reporting reliable and consistent methods of feature extraction, and consider prospective, well-controlled methodologic designs to improve the quality of the evidence.

## Figures and Tables

**Table 1:** Studies reporting on SUVmax of the primary breast tumour in relation to axillary lymph node status ^(a)^.

	Mean or Median Tumour SUVmax in ALN+ vs. ALN− Cases	Multivariable Analysis of Tumour SUVmax [Other Variables Included in the Model]	ROC Analysis on Tumour SUVmax
An 2017 [21]	4.9 vs. 4.6 Not significant	-	-
Can 2019 [22]	4.1 vs. 3.9 Not significant	-	-
Challa 2013 [25]	6.6 vs. 6.7 p not reported	-	-
Chen 2022 [26]	6.68 vs. 6.31 Not significant	-	-
Erol 2021 [27]	7.99 vs. 8.17 Not significant	-	-
Jung 2016 [28]	4.93 vs. 3.22 *p* < 0.0001	With Cutoff 2.8, OR: 4 (*p* = 0.04) [age, SUVmax of ALN (cutoff: 0.72), Size, LVI, Nuclear grade, Histologic grade, HER2]	Cutoff: 2.8, AUC: 67.7%, accuracy 67.7%, sensitivity 63.2%, specificity 65%
Karabacak 2023 [29]	5 vs. 3.8 *p* = 0.042	-	Cutoff 4.05, AUC: 61.8%, accuracy 57%, sensitivity 58%, specificity 55%
Karan 2016 [30]	N0 patients: 4.30 N1 patients: 6.18 N2 patients: 10.80 N3 patients: 10.53 *p* = 0.015	-	-
Kim 2015 [31]	8.6 vs. 6.2 *p* < 0.001	Cutoff: 4.25, OR: 3.497 (2.245–5.446), *p* < 0.001. [Age, Tumour size > 2 cm, Histological grade (grade 3 vs. 2), ER Status, PR status, HER2 status, P53 status, Ki67 status, LVI, Histology (ductal versus other)]	-
Kong 2021 [32]	N0: 3.44 Low burden: 6.04 High burden: 5.82 Not significant	-	-
Monzawa 2009 [35]	4.0 vs. 3.3 Not significant	-	-
Ozen 2024 [36]	6.56 vs. 5.79 Not significant	-	-
Ozkan 2019 [39]	-	-	Cutoff 1.79, AUC 84.7%, accuracy 84%, sensitivity 79%, specificity 93%
Pahk 2020 [40]	3.17 vs. 2.35 *p* = 0.012	Cutoff: determined based on molecular subtype. Not significant. [BMI, T stage, LVI, visceral/subcutaneous fat ratio]	-
Song 2021 [42]	8.6 vs. 9.4 Not significant	-	-
Song 2017 [43]	7.9 vs. 7.9 Not significant	No significant results with a cutoff of 3.9. If cutoff determined by molecular subtype, OR 4.87, *p* = 0.0037 [LVI, tumour size, SUVmax cutoff 3.9, nodal uptake]	Cutoff 3.9, AUC 59.7%
Taira 2009 [45]	4.61 vs. 3.85 Not significant	-	-
Yoo 2018 [46]	5.32 vs. 3.97 *p* = 0.022	No significant results [tumour size, TLG]	Cutoff 3.11, AUC 63.6%

^(a)^ SUVmax: maximum standardized uptake value; ALN: axillary lymph node; LVI: lymphovascular invasion; AUC: area under the receiver operating characteristics curve; OR: odds ratio, Nx refers to pathologic nodal stage.

**Table 2:** Results of receiver-operating characteristic analysis in two studies investigating radiomic features in relation to axillary lymph node status ^(a)^.

	Sensitivity	Specificity	PPV	NPV	Accuracy	AUC
Chen 2022 [26] RF Model	-	-	-	-	81.2% (65.3–93.9%)	81.7% (66.1–92.9%)
Chen 2022 [26] SGD Model	-	-	-	-	74.5% (50–87.5%)	77.5% (50.6–89.2%)
Chen 2022 [26] KNN Model	-	-	-	-	78.5% (64.3–89.3%)	79.5% (64.5–88.5%)
Chen 2022 [26] SVM Model	-	-	-	-	75.6% (60.7–89.3%)	78.3% (66.0–87.7%)
Song 2021 [42]	90.9%	71.4%	71.4%	90.9%	80%	89.0%

^(a)^ PPV: positive predictive value, NPV: negative predictive value, AUC: area under the receiver operating characteristic curve.

## Supplementary Materials

The following supporting information can be downloaded at: https://www.mdpi.com/article/10.3390/curroncol32060300/s1, Supplemental S1: Literature Search Strategies; Supplemental S2: Tailored QUADAS-2 tool.

## Abbreviations

The following abbreviations are used in this manuscript:

ALN	Axillary lymph node
SUV	Standardized uptake value
MTV	Metabolic tumour volume
TLG	Total lesion glycolysis
ROI	Region of interest
ROC	Receiver-operating characteristics

## Appendix A

**Table A1:** Characteristics of included studies ^(a)^.

	Study Type	Country	Size (Patients)	Age (Mean or Median)	Clinical Stage	Axillary Nodal Status	Details of PET/CT Acquisition	Region of Interest (Method of Delineation)	Features
An 2017 [21]	Retrospective cohort	South Korea	173	49	Not specified	128 ALN−, 45 ALN+	Dosage: 5 MBq/kg Blood glucose: <150 mg/dL Acquisition parameters: 60 min uptake, 3 min/frame, 7–8 frames, 600 mm FOV Reconstruction parameters: OSEM algorithm, 2 iterations, 20 subsets, slice thickness 3.27 mm	Breast tumour (semi-automatic)	Semiquantitative (SUVmax, SUVmean, MTV, TLG)
Can 2019 [22]	Retrospective cohort	Turkey	129	49	Not specified	61 ALN−, 68 ALN+	Dosage: 5.5 MBq/kg Blood glucose: <=140 mg/dL Acquisition parameters: 60 min uptake, 3 min/bed position, 15.5 cm FOV Reconstruction parameters: OSEM algorithm, 2 iterations, 8 subsets, FWHM 5 mm	Breast tumour (manual)	Semiquantitative (SUVmax, MTV, TLG)
Cetindag 2023 [23]	Retrospective cohort	Turkey	67	56	Not specified	33 ALN−, 34 ALN+	Dosage: Not specified Blood glucose: <150 mg/dL Acquisition parameters: Not specified Reconstruction parameters: Not specified	Most prominent lymph node (manual)	Semiquantitative (SUVmax)
Chae 2009 [24]	Retrospective cohort	South Korea	108	49	77 T1, 29 T2, 2 T3 18 N1, 10 N2, 5 N3	75 ALN−, 33 ALN+	Dosage: 5 mCi Blood glucose: <120 mg/dL Acquisition parameters: 60 min uptake Reconstruction parameters: Not specified	Axilla (not specified)	Semiquantitative (SUVmax)
Challa 2013 [25]	Prospective cohort	India	37	51	T1 or T2 with N0	21 ALN−, 16 ALN+	Dosage: 370 Mbq Blood glucose: <140 mg/dl Acquisition parameters: 45–60 min uptake, 2–3 min/bed position Reconstruction parameters: OSEM algorithm, 2 iterations, 8 subsets, slice thickness 1.5 mm, matrix of 128 × 128 pixels.	Breast tumour and axilla (manual)	Semiquantitative (SUVmax)
Chen 2022 [26]	Retrospective cohort	China	180	55	cN0	28 ALN−, 152 ALN+	Dosage: 0.10–0.15 mCi/kg Blood glucose: <11.1 mmol/L Acquisition parameters: 60 min uptake, 2 min/bed position, 8 bed positions Reconstruction parameters: OSEM algorithm, 6 mm FWHM, voxel size 5.3 × 5.3 × 2.5 mm	Breast tumour (manual)	Radiomics Features of first order, shape, and texture (1562 PET features and 1562 CT features, 3124 PET/CT features total)
Erol 2021 [27]	Retrospective cohort	Turkey	143	52	Not specified	65 ALN−, 78 ALN+	Dosage: Not specified Blood glucose: <150 mg/dL Acquisition parameters: 60 min uptake Reconstruction parameters: Not specified	Breast tumour (manual)	Semiquantitative (SUVmax, SUVmean, MTV, TLG)
Jung 2016 [28]	Retrospective cohort	South Korea	428	50	Not specified	261 ALN−, 167 ALN+	Dosage: 370–550 MBq Blood glucose: <130 mg/dL Acquisition parameters: 60 min uptake, 2 min/bed position, 7–8 bed positions Reconstruction parameters: Not specified	Breast tumour and axilla (manual)	Semiquantitative (SUVmax)
Karabacak 2023 [29]	Retrospective cohort	Turkey	104	57	Not specified ^(b)^	42 ALN−, 62 ALN+	Dosage: 0.14 mCi/kg Blood glucose: <180 mg/dL Acquisition parameters: 60 min uptake, 3 min/bed position Reconstruction parameters: Not specified	Breast tumour and axilla (semi-automatic)	Semiquantitative (SUVmax)
Karan 2016 [30]	Retrospective cohort	Turkey	70	47	Not specified	19 ALN−, 51 ALN+	Dosage: 297–370 MBq Blood glucose: <150 mg/dL Acquisition parameters: 60 min uptake, 2.5 min/bed position, 6–7 bed positions Reconstruction parameters: Not specified	Breast tumour (automatic)	Semiquantitative (SUVmax)
Kim 2015 [31]	Retrospective cohort	South Korea	671	52	Not specified	392 ALN−, 279 ALN+	Dosage: 5.2 MBq/kg Blood glucose <120 mg/dL Acquisition parameters: 60 min uptake Reconstruction parameters: Iterative algorithms 2 iterations, 21 subsets, 168 × 168 image matrices	Breast tumour (manual)	Semiquantitative (SUVmax)
Kong 2021 [32]	Retrospective cohort	South Korea	221	49	T1 174, T2 50	161 ALN−, 63 ALN+	Dosage: 4.44 MBq/kg Blood glucose: <160 mg/dL Acquisition parameters: 60 min, 3–4 min/frame, 7–8 frames Reconstruction parameters: Iterative algorithms 2 iterations, 8 subsets. FWHM 5.0 mm	Breast tumour (not specified)	Semiquantitative (SUVmax)
Kulahci 2021 [33]	Retrospective cohort	Turkey	113	52	Not specified	64 ALN−, 49 ALN+	Dosage: 5 MBq/kg Blood glucose: <200 mg/dL Acquisition parameters: 60 min uptake, 2–4 min/bed position Reconstruction parameters: Not specified	Axilla (manual)	Semiquantitative (SUVmax)
Kutluturk 2019 [34]	Retrospective cohort	Turkey	232	51	Mean clinical tumour size: 2.4 cm	68 ALN−, 164 ALN+	Dosage: 0.1 mg/kg Blood glucose: Not specified Acquisition parameters: 60 min uptake Reconstruction parameters: OSEM algorithm	Breast tumour and axilla (not specified)	Semiquantitative (SUVmax)
Monzawa 2009 [35]	Retrospective cohort	Japan	50	59	Stage I in 34 patients, II in 15 patients, and III in 1 patient	35 ALN−, 15 ALN+	Dosage: 185 mBq Blood glucose: Not specified Acquisition parameters: 50–60 min uptake, 2 min/bed position Reconstruction parameters: 128 × 128 matrix, section thickness of 3.27 mm	Breast tumour (manual)	Semiquantitative (SUVmax)
Ozen 2024 [36]	Retrospective cohort	Turkey	40	52	Stage 1A: 12, 1B 1, 2A 10, 2B 8, 3A 6, 3C 3	18 ALN−, 22 ALN+	Dosage: 2.5 MBq/kg Blood glucose: Not specified Acquisition parameters: 60 min uptake, 3 min/bed position Reconstruction parameters: Iterative algorithm	Breast tumour, axilla, normal parenchyma, liver (manual)	Semiquantitative (SUVmax, tumour to parenchyma SUVmax ratio, tumour to liver SUVmax ratio, axilla to parenchyma SUVmax ratio, axilla to liver SUVmax ratio)
Ozer 2021 [37]	Retrospective cohort	Turkey	90	55	Early breast cancer	36 ALN−, 54 ALN+	Dosage: 369 MBq Blood glucose: <200 mg/dL Acquisition parameters: 60 min uptake Reconstruction parameters: Thinned slices up to 2 mm, using xSHARP technology and Richardson-Lucy maximum likelihood algorithm	Breast tumour and axilla (Manual)	Semiquantitative (SUVmax)
Ozgur Aytac 2015 [38]	Retrospective cohort	Turkey	273	50	131 T1, 142 T2	105 ALN−, 168 ALN+	No details specified	Axilla (not specified)	Semiquantitative (SUVmax)
Ozkan 2019 [39]	Retrospective cohort	Turkey	192	51	Stage IB to IIIA	80 ALN−, 112 ALN+	Dosage: 3.7–5.5 MBq/kg Blood glucose: <150 g/mL Acquisition parameters: 60 min uptake Reconstruction parameters: Not specified	Breast tumour and axilla (manual)	Semiquantitative (SUVmax)
Pahk 2020 [40]	Retrospective cohort	South Korea	173	62	Not specified	108 ALN−, 65 ALN+	Dosage: 5.29 MBq/kg Blood glucose: Not specified Acquistion parameters: 60 min uptake, 1 min/bed position, 9 bed positions, 18 cm axial FOV, 4.4 mm spatial resolution, Reconstruction parameters: 3D row-action maximum likelihood algorithm	Breast tumour, visceral (V) and subcutaneous (S) adipose tissue (manual)	Semiquantitative (SUVmax, V/S SUVmax ratio)
Park 2014 [41]	Retrospective cohort	South Korea	136	50	Not specified	66 ALN−, 70 ALN+	Dosage: 7.4 MBq/kg Blood glucose: <7.2 mmol/L Acquisition parameters: 60 min uptake, 3.5 min/bed position, 5–6 bed positions, 16.2 cm axial FOV Reconstruction parameters: OSEM algorithm, 2 iterations, 8 subsets	Breast tumour and axilla (manual)	Semiquantitative (SUVmax, lymph node to tumour SUVmax ratio)
Song 2021 [42]	Retrospective cohort	South Korea	100	Not specified	Not specified ^(b)^	57 ALN−, 43 ALN+	Dosage: 5.5 MBq/kg Blood glucose: <8.3 mmol/L Acquisition parameters: 60 min uptake, 3 min/bed position Reconstruction parameters: OSEM algorithm	Breast tumour (automatic)	Radiomics (792 radiomics features)
Song 2017 [43]	Retrospective cohort	South Korea	128	53	Not specified ^(b)^	76 ALN−, 52 ALN+	Dosage: 5.5 MBq/kg Blood glucose: <8.3 mmol/L Acquisition parameters: 60 min uptake, 3 min/bed position Reconstruction parameters: OSEM algorithm	Breast tumour (manual)	Semiquantitative (SUVmax)
Sun 2016 [44]	Retrospective cohort	South Korea	196	54	cT1-3 N0-3 TNM stage I: 89, II: 88, III: 19	131 ALN−, 65 ALN+	Dosage: 555 MBq/kg Blood glucose: <200 mg/dL Acquisition parameters: 50 min uptake, 2.5 min/bed position, 6–8 bed positions Reconstruction parameters: Not specified	Breast tumour and axilla (manual)	Semiquantitative (SUVmax, ALN SUVmax ratio)
Taira 2009 [45]	Retrospective cohort	Japan	90	55	cN0	65 ALN−, 27 ALN+	Dosage: 3 MBq/kg Blood glucose: Not specified Acquisition parameters: 60 min uptake Reconstruction parameters: Not specified	Breast tumour and axilla (manual)	Semiquantitative (SUVmax)
Yoo 2018 [46]	Retrospective cohort	South Korea	135	54	cN0	104 ALN−, 31 ALN+	Dosage: 3.7 MBq/kg Blood glucose: <140 mg/dL Acquisition parameters: 60 min uptake Reconstruction parameters: OSEM algorithm, 2 iterations, 21 subsets, 200 × 200 matrices, 3.4 × 3.4 mm pixel size, slice thickness 3.0 mm	Breast tumour (semi-automatic)	Semiquantitative (SUVmax, MTV, TLG)
Zhang 2021 [47]	Retrospective cohort	China	40 patients, 209 lymph nodes	56	All stage III (N2) disease (pathologic)	112 ALN−, 97 ALN+ (Individual lymph nodes)	Dosage: 4.81 MBq/kg Blood glucose: <8.2 mmol/L Acquisition parameters: 60–70 min uptake, 2 min/body position, 6–7 positions Reconstruction parameters: 5 mm slice thickness	ALNs (manual)	Semiquantitative (SUVmax, early phase of dual-point PET-CT)
Zhang 2014 [48]	Retrospective cohort	China	164	45	cN0	123 ALN−, 41 ALN+	Dosage: 5.3 MBq/kg ± 10% Blood glucose: <11.0 mmol/L Acquisition parameters: 60 min uptake, 5–7 bed positions Reconstruction parameters: OSEM algorithm, 2 iterations, 28 subsets. FWHM 8 mm	Axilla (manual)	Semiquantitative (SUVmax)

^(a)^ ALN: axillary lymph node, FOV: field of view, FWHM: full width at half maximum, OSEM: ordered-subset expectation maximization, SUVmax: maximum standardized uptake value, MTV: metabolic tumour volume, TLG: total lesion glycolysis. ^(b)^ Though some staging information was provided, it was unclear what type of staging was described (i.e., clinical vs. pathologic).

**Table A2:** Studies reporting on SUVmax of the axilla/ALNs in relation to ALN status ^(a)^.

	Mean or Median ALN SUVmax in ALN+ vs. ALN− Cases	Multivariable Analysis of ALN SUVmax [Other Variables Included in the Model]	ROC Analysis on ALN SUVmax
Cetindag 2023 [23]	6.3 vs. 1.6 *p* = 0.001	-	-
Chae 2009 [24]	-	-	Cutoff 0.64 Accuracy 73.2%, sensitivity 48.5%, specificity 84%
Challa 2013 [25]	-	-	Cutoff 0.5 AUC 75.4%, sensitivity 56%, specificity 90%
Jung 2016 [28]	-	Cutoff 0.72, OR 5.37, *p* < 0.005 [age, tumour SUVmax, size, lymphatic invasion, nuclear grade, histologic grade, HER2]	Cutoff 0.72 AUC 78.3%, accuracy 65%, sensitivity 86%, specificity 78%
Karabacak 2023 [29]	2.2 vs. 1.0 *p* < 0.001	-	Cutoff 1.25 AUC 77.6%, accuracy 74%, sensitivity 61%, specificity 93%
Kulahci 2021 [33]	-	-	Cutoff: 1.84 AUC 75.6%, sensitivity 53%, specificity: 86%
Kutluturk 2019 [34]	-	Cutoff 3.2, OR 15.7, *p* < 0.001 [largest tumour size]	-
Ozer 2021 [37]	ALN+ nodal SUVmax was 1.939 times higher than ALN− nodal SUVmax (*p* = 0.001)	-	Cutoff 1.1 Sensitivity 67%, specificity 69%
Ozgur Aytac 2015 [38]		Cutoff 2.5, OR 14, *p* = 0.005 [tumour stage, hormone receptor status, histology type, age, SUVmax cutoff of 1]	Cutoff 1.6 Sensitivity: 56%, specificity: 83% Cutoff 2.5 AUC 71.5%, sensitivity 42%, specificity 90%
Park 2014 [41]	-	-	Cutoff 2.1 AUC 70.5%, accuracy 70%, sensitivity 47%, specificity 94%
Sun 2016 [44]	-	Cutoff of 1 was significant for ALN metastases with *p* < 0.001 (details not specified). [primary tumour SUVmax, SUVmax ratio]	Cutoff 1 AUC 74.6%, sensitivity 54%, specificity 94%
Taira 2009 [45]	-	-	Cutoff 2.0 Sensitivity 37%, specificity 99%
Zhang 2021 [47]	SUVmax of matched lymph nodes: 5.45 vs. 2.79 *p* < 0.001	-	Cutoff 4.29 AUC 96.1%, sensitivity 82%, specificity 100%
Zhang 2014 [48]	-	-	Cutoff 1.5 Accuracy 80%, sensitivity 46%, specificity 91%

^(a)^ SUVmax: maximum standardized uptake value; ALN: axillary lymph node; AUC: area under the receiver operating characteristics curve.
