# Supplementary material for: 18F-FDG PET/CT Semiquantitative and Radiomic Features for Assessing Pathologic Axillary Lymph Node Status in Clinical Stage I–III Breast Cancer Patients: A Systematic Review
Source: Curr Oncol. 2025 May 23;32(6):300. doi: 10.3390/curroncol32060300 (PMC12192514; doi:10.3390/curroncol32060300)
Supplement: Supplementary file 1 [file curroncol-32-00300-s001.zip › Supplemental S1.pdf]

Supplemental for "18F-FDG PET/CT semiquantitative and radiomic features for assessing pathologic axillary lymph node status in clinical stage I-III breast cancer patients: A systematic review"

Anna Hwang, Sana Rashid, Selina Shi, Ciara Blew, Mark Levine, Ashirbani Saha

Literature Search Strategies

Medline [Ovid] Search Strategy

1. Tomography, Emission-Computed / or (positron adj emission adj tomography).ti,ab. or PET.ti,ab. or PET-FDG.ti,ab. Or Fluorodeoxyglucose F18/ or 18f fluorodeoxyglucose.ti,ab. or 18fdg.ti,ab. or 2-fluoro-2-deoxyglucose.ti,ab. or 2-fluoro-2-deoxyglucose.ti,ab. or 18f-fdg.ti,ab. or fluorine-18-flourodeoxyglucose.ti,ab. or fluorine-18fluorodeoxyglucose.ti,ab. or flourine-18-fluorodeoxyglucose.ti,ab. or flourine-18-flourodeoxyglucose.ti,ab. or fluorine-18-fluordeoxyglucose.ti,ab. or positron emission tomography/ or PET-CT.ti,ab. or PET$CT.ti,ab. or FDG-PET*.ti.ab.
2. exp Breast neoplasms/ or breast.ti,ab.
3. Neoplasm staging/ or (stage or staging or metasta$).ti,ab.
4. (axillary lymph node or nodal status or node status).mp.
5. 3 or 4
6. (radiomic or radiomics or texture or textural features or textural analysis or histogram* or SUV or texture* or radiologic features or imaging features or computer assisted or computer extracted or radiogenomic*).mp.
7. (standard uptake value* or standardized uptake value* or metabolic parameter* or metabolic tumor volume or metabolic tumour volume or tumour lesion glycolysis or tumor lesion glycolysis).mp.
8. 6 or 7
9. 1 and 2 and 5 and 8
10. limit 9 to (abstracts and English language)

Central [Cochrane] Search Strategy

1. MeSH descriptor: [Positron-Emission Tomography] explode all trees
2. MeSH descriptor: [Fluorodeoxyglucose F18] explode all trees
3. PET:ti,ab or PET-FDG:ti,ab or 'fluorodeoxyglucose:ti,ab or 18fdg:ti,ab or PET-CT:ti,ab or PETCT:ti,ab or "Positron Emission Tomography" or *deoxyglucose:ti,ab
4. MeSH descriptor: [Breast Neoplasms] explode all trees
5. breast.ti,ab
6. MeSH descriptor: [Neoplasm Staging] explode all trees
7. Late stage:ti,ab or early stage:ti,ab or stage:ti,ab or staging:ti,ab or II*:ti,ab or III*:ti,ab or metasta*:ti,ab
8. #1 or #2 or #3
9. #4 or #5
10. #6 or #7
11. #9 and #10
12. radiomic*:ti,ab or textur*:ti,ab or features*:ti,ab or indices*ti,ab or histogram:ti,ab or computer:ti,ab or SUV:ti,ab or radiogenomic:ti,ab
13. "standard uptake value":ti, ab or "standardized uptake value":ti,ab or "metabolic parameter":ti,ab or "metabolic tumor volume":ti,ab or "MTV":ti,ab or "tumor lesion glycolysis":ti,ab or "TLG":ti,ab
14. #12 or #13
15. axillary lymph node:ti,ab or nodal status:ti,ab or node status:ti,ab
16. #10 or #15
17. #9 and #16
18. #8 and #17 and #14

Embase [Ovid] Search Strategy

1. Tomography, Emission-Computed / or (positron adj emission adj tomography).ti,ab. or PET.ti,ab. or PET-FDG.ti,ab. Or Fluorodeoxyglucose F18/ or 18f fluorodeoxyglucose.ti,ab. or 18fdg.ti,ab. or 2-fluoro-2-deoxyglucose.ti,ab. or 2-fluoro-2-deoxyglucose.ti,ab. or 18f-fdg.ti,ab. or fluorine-18-flourodeoxyglucose.ti,ab. or fluorine-18fluorodeoxyglucose.ti,ab. or flourine-18-fluorodeoxyglucose.ti,ab. or flourine-18-flourodeoxyglucose.ti,ab. or fluorine-18-fluordeoxyglucose.ti,ab. or positron emission tomography/ or PET-CT.ti,ab. or PET$CT.ti,ab. or FDG-PET*.ti.ab.
2. exp Breast neoplasms/ or breast.ti,ab.
3. Neoplasm staging/ or (stage or staging or metasta$).ti,ab.
4. (axillary lymph node or nodal status or node status).mp.
5. 3 or 4
6. (radiomic or radiomics or texture or textural features or textural analysis or histogram* or SUV or texture* or radiologic features or imaging features or computer assisted or computer extracted or radiogenomic*).mp.
7. (standard uptake value* or standardized uptake value* or metabolic parameter* or metabolic tumor volume or metabolic tumour volume or tumour lesion glycolysis or tumor lesion glycolysis).mp.
8. 6 or 7
9. 1 and 2 and 5 and 8
10. limit 9 to (abstracts and English language)
11. limit 10 to (article or article in press or conference paper or “preprint (unpublished, non-peer reviewed)”)
