# Supplementary material for: 18F-FDG PET/CT Semiquantitative and Radiomic Features for Assessing Pathologic Axillary Lymph Node Status in Clinical Stage I–III Breast Cancer Patients: A Systematic Review
Source: Curr Oncol. 2025 May 23;32(6):300. doi: 10.3390/curroncol32060300 (PMC12192514; doi:10.3390/curroncol32060300)
Supplement: Supplementary file 1 [file curroncol-32-00300-s001.zip › Supplemental S2.pdf]

Supplemental for "18F-FDG PET/CT Semiquantitative and Radiomic Features for Assessing Pathologic Axillary Lymph Node Status in Clinical Stage I–III Breast Cancer Patients: A Systematic Review"

Anna Hwang, Sana Rashid, Selina Shi, Ciara Blew, Mark Levine, Ashirbani Saha

Tailored QUADAS-2 Tool

Phase 1: State the review question:

Patients (setting, intended use of index test, presentation, prior testing):<br>- Females with newly diagnosed clinical stage I-III breast cancer who underwent PET/CT for initial staging and in whom sentinel lymph node biopsy and/or axillary node dissection was performed prior to any therapy.
Index tests:<br>- Semi-quantitative and quantitative features extracted from staging PET/CT
Reference Standard:<br>- Histopathology from sentinel lymph node biopsy and/or axillary node dissection
Target Condition<br>- Comparison of semiquantitative and quantitative features with pathologically confirmed axillary lymph node status

Phase 2: Draw a flow diagram for the primary study

See example on the last page.

Phase 3: Risk of bias and applicability judgements

QUADAS-2 is structured so that 4 key domains are each rated in terms of the risk of bias and the concern regarding applicability to the research question (as defined above). Each key domain has a set of signalling questions to help reach the judgments regarding bias and applicability.

Domain 1: Patient Selection

A. Risk of Bias

Describe the methods of patient selection

- Was a consecutive or random sample of patents enrolled? Yes/No/Unclear
- Was a case-control design avoided? Yes/No/Unclear
- Did the study avoid excluding patients based on presence or absence of clinical findings (including symptomatology, palpability, conventional imaging)? Yes/No/Unclear

- Did the study avoid excluding patients based on age, risk factors, tumour biomarkers? Yes/No/Unclear
- Did the study avoid excluding patients due to low FDG uptake on PET/CT? Yes/No/Unclear
- Did the authors clearly specify whether the patients had neoadjuvant therapy prior to surgery? Yes/No/Unclear

Could the selection of patients have introduced bias? RISK: LOW/HIGH/UNCLEAR

Low	If the answer to all additional questions is 'Yes', then risk of bias can be considered low
High	If the answer to any of the signalling questions is 'No', there is a potential for bias. If one or more of the answers is 'No', the judgement could still be low risk of bias, but specific reasons why the risk of bias can be considered low should be provided
Unclear	If relevant information is missing for all or some of the signalling questions, and none of the answers to signalling questions is judged to put the study at high risk of bias

B. Concerns regarding applicability

Describe included patients (prior testing, presentation, intended use of index test and setting):

- Did the authors clearly specify clinical stage of their population or did they clearly specify that distant metastases were excluded? Yes/No/Unclear
- Did the authors specify if only women were included? Yes/No/Unclear

Is there concern that the included patients do not match the review question or that the patient population is poorly described? **CONCERN: LOW/HIGH/UNCLEAR**

Low	If the spectrum of participants (inclusion and exclusion criteria, setting, prior testing) matches the pre-stated requirements in the review question
High	If the spectrum of participants does not fully match the pre- stated requirements in the review question
Unclear	If there is insufficient information available to make a judgement about applicability

Domain 2: Index Test(s)

Semiquantitative and Quantitative Features extracted

A. Risk of Bias

Describe the methods by which semi-quantitative and/or quantitative features were obtained:

- Were the people involved in feature extraction blinded to the results of sentinel lymph node biopsy and axillary dissection? Yes/No/Unclear
- Was the method of PET/CT image acquisition clearly described? Yes/No/Unclear
- If the region of interest was drawn manually or semi-automatically with manual correction, was this done by an experienced nuclear medicine physician? If the region of interest was determined semi-automatically, was an SUV threshold pre-specified? If the region of interest was determined automatically, was the algorithm used specified? Yes/No/Unclear
- If segmentation was performed manually or semi-automatically, was intra-observer and inter-observer reliability tested? Yes/No/Unclear
- For semi-quantitative features, did the authors clearly describe how these were obtained including thresholds (if applicable) used for calculations? For quantitative features, did the authors specify the software or add methodological details used to extract the features? Yes/No/Unclear

Could the conduct or interpretation of the index test have introduced bias? RISK: LOW/HIGH/UNCLEAR

Low	If the answer to all additional questions is 'Yes', then risk of bias can be considered low
High	If the answer to any of the signalling questions is 'No', there is a potential for bias. If one or more of the answers is 'No', the judgement could still be low risk of bias, but specific reasons why the risk of bias can be considered low should be provided
Unclear	If relevant information is missing for all or some of the signalling questions, and none of the answers to signalling questions is judged to put the study at high risk of bias

B. Concerns regarding applicability
Is there concern that the index test (semiquantitative/quantitative features), its conduct, or interpretation differ from the review question? CONCERN: LOW/HIGH/UNCLEAR

Low	If the index test technology and the way it was obtained and interpreted in the study match the pre-stated requirements in the review question
High	If there are differences in index test technology, execution and interpretation between the study and the review question
Unclear	If there is insufficient information available to make a judgement about applicability for this domain

Domain 3: Reference Standard

A. Risk of Bias

Describe the reference standard (sentinel lymph node biopsy and/or axillary dissection) and how it was conducted and interpreted:

- Did the authors clearly describe the methods by which the reference standard was obtained and interpreted? Yes/No/Unclear
- Is the reference standard likely to correctly classify the target condition (axillary lymph node stage)? Yes/No/Unclear
- Were the results of sentinel lymph node biopsy and/or axillary dissection interpreted without knowledge of the results of the PET/CT radiomic features? Yes/No/Unclear

Could the reference standard, its conduct, or its interpretation have introduced bias? RISK: LOW/HIGH/UNCLEAR

Low	If the answer to all additional questions is 'Yes', then risk of bias can be considered low
High	If the answer to any of the signalling questions is 'No', there is a potential for bias. If one or more of the answers is 'No', the judgement could still be low risk of bias, but specific reasons why the risk of bias can be considered low should be provided
Unclear	If relevant information is missing for all or some of the signalling questions, and none of the answers to signalling questions is judged to put the study at high risk of bias

B. Concerns regarding applicability
Is there concern that the target condition as defined by the reference standard does not match the review question? CONCERN: LOW/HIGH/UNCLEAR

Low	If the reference standard, as used in the study, detects the target condition defined in the review question
High	If the reference standard, as used in the study, does not detect the same (form of) target condition as defined in the review question
Unclear	If there is insufficient information available to make a judgement about applicability for this domain

<u>Domain 4</u>: Flow and Timing

A. Risk of Bias

Describe any patients who did not receive the index test(s) and/or reference standard or who were excluded from the 2x2 table (refer to flow diagram):

Describe the time interval and any interventions between index test(s) and reference standard:

- Was there an appropriate interval between PET/CT and sentinel lymph node biopsy / axillary dissection? (i.e., can we be reasonably assured that there was no significant change in the axillary region between initial imaging and surgery)? Yes/No/Unclear
- Did the patients all receive the same reference standard? Yes/No/Unclear
- Were all patients included in the analysis? Yes/No/Unclear

Could the patient flow have introduced bias? RISK: LOW/HIGH/UNCLEAR

Low	If the answer to all additional questions is 'Yes', then risk of bias can be considered low
High	If the answer to any of the signalling questions is 'No', there is a potential for bias. If one or more of the answers is 'No', the judgement could still be low risk of bias, but specific reasons why the risk of bias can be considered low should be provided
Unclear	If relevant information is missing for all or some of the signalling questions, and none of the answers to signalling questions is judged to put the study at high risk of bias

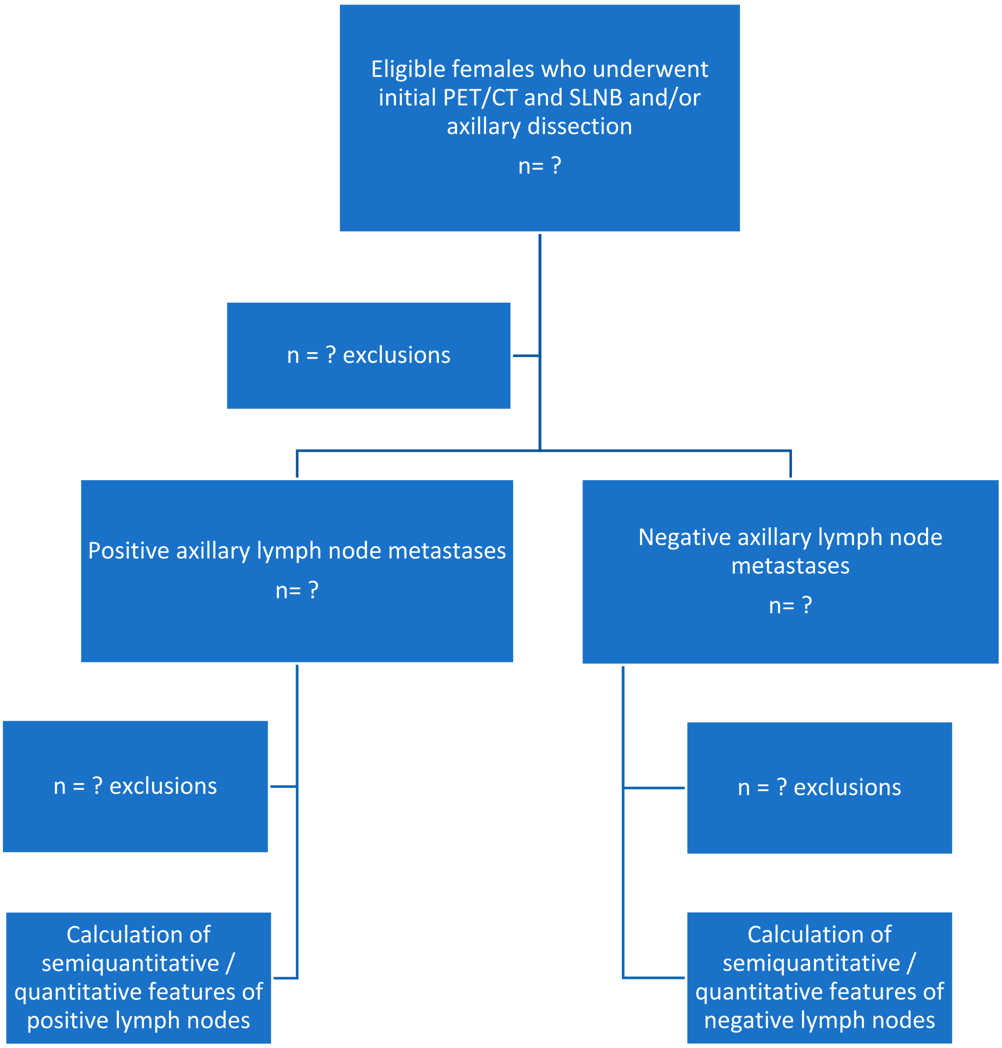

Figure S1: Example flow diagram of a primary study
